# Secondary syphilis in HIV with CD8-predominant epidermotropic infiltrate mimicking cutaneous T-cell lymphoma

**DOI:** 10.1016/j.idcr.2026.e02765

**Published:** 2026-09-26

**Authors:** Aída S. Ramírez-González, Felipe J. Bustos-Rodríguez, Luz A. González-Hernández, Jaime F. Andrade-Villanueva, Luis H. Sat-Dávila, Pedro Martínez-Ayala

**Affiliations:** a Departamento de Dermatología, Hospital Civil de Guadalajara “Fray Antonio Alcalde”, Guadalajara, Mexico; b Departamento de Microbiología y Patología, Centro Universitario de Ciencias de la Salud, Universidad de Guadalajara, Guadalajara, Mexico; c Unidad de VIH, Hospital Civil de Guadalajara “Fray Antonio Alcalde”, Guadalajara, Mexico

**Keywords:** Secondary syphilis, Cutaneous T-cell lymphoma, Mycosis fungoides, HIV, Treponema pallidum, CD8, Pseudolymphoma, Case report

## Abstract

**Background:**

Syphilis and mycosis fungoides are both classically referred to as “great imitators” of skin disease, and their differential diagnosis is particularly challenging in people living with HIV (PLWH). Secondary syphilis may produce a plasma-cell-poor, epidermotropic, CD8-predominant, highly proliferative infiltrate that closely resembles aggressive cutaneous T-cell lymphoma (CTCL).

**Case presentation:**

A 49-year-old man who has sex with men (MSM), with poorly controlled HIV (CD4 340 cells/µL, viral load 6220 copies/mL, ART discontinued two years earlier), presented with widespread coalescing copper-red macules, papules and infiltrated plaques in a corymbriform-like configuration. A skin biopsy and syphilis serology were ordered simultaneously as part of the initial diagnostic work-up. The nontreponemal test (VDRL) was strongly reactive at 1:128 and the treponemal test was reactive; however, biopsy demonstrated an epidermotropic CD3⁺/CD8⁺ infiltrate with Ki-67 ≈ 80% and admixed plasma cells, initially interpreted as suggestive of an aggressive epidermotropic CD8⁺ CTCL, and the strongly reactive serology was initially regarded as an incidental coinfection. Recognition of the plasma cells and of features atypical for CTCL (endothelial swelling, absence of necrotic ulcerated nodules, sub-acute course) prompted a Warthin–Starry stain on the same biopsy, which identified *Treponema pallidum* in situ and secured the diagnosis of secondary syphilis. Intramuscular benzathine penicillin G (2.4 million IU weekly × 3, per institutional practice at that time) and reinitiation of antiretroviral therapy led to complete resolution of the dermatosis at six months, with residual post-inflammatory hyperpigmentation.

**Conclusions:**

An epidermotropic CD8-predominant infiltrate with high Ki-67 in an HIV-coinfected patient does not exclude secondary syphilis. Careful integration of clinical, histopathologic, immunohistochemical and serologic data — and in situ demonstration of *T. pallidum* (anti-treponemal immunohistochemistry, Warthin–Starry, or lesional PCR) — is essential to resolve the clinicopathologic discordance and avoid unnecessary oncologic therapy.

## Highlights

• Secondary syphilis mimicked aggressive CD8 + cutaneous T-cell lymphoma in HIV.
• VDRL 1:128 was overlooked amid striking histopathology — cognitive anchoring.
• Warthin–Starry stain confirmed T. pallidum in situ within the skin biopsy.
• Benzathine penicillin plus reinitiated ART led to full resolution at 6 months.
• An epidermotropic CD8 + Ki-67-high infiltrate does not exclude syphilis.

## Graphical Abstract

**Figure:**
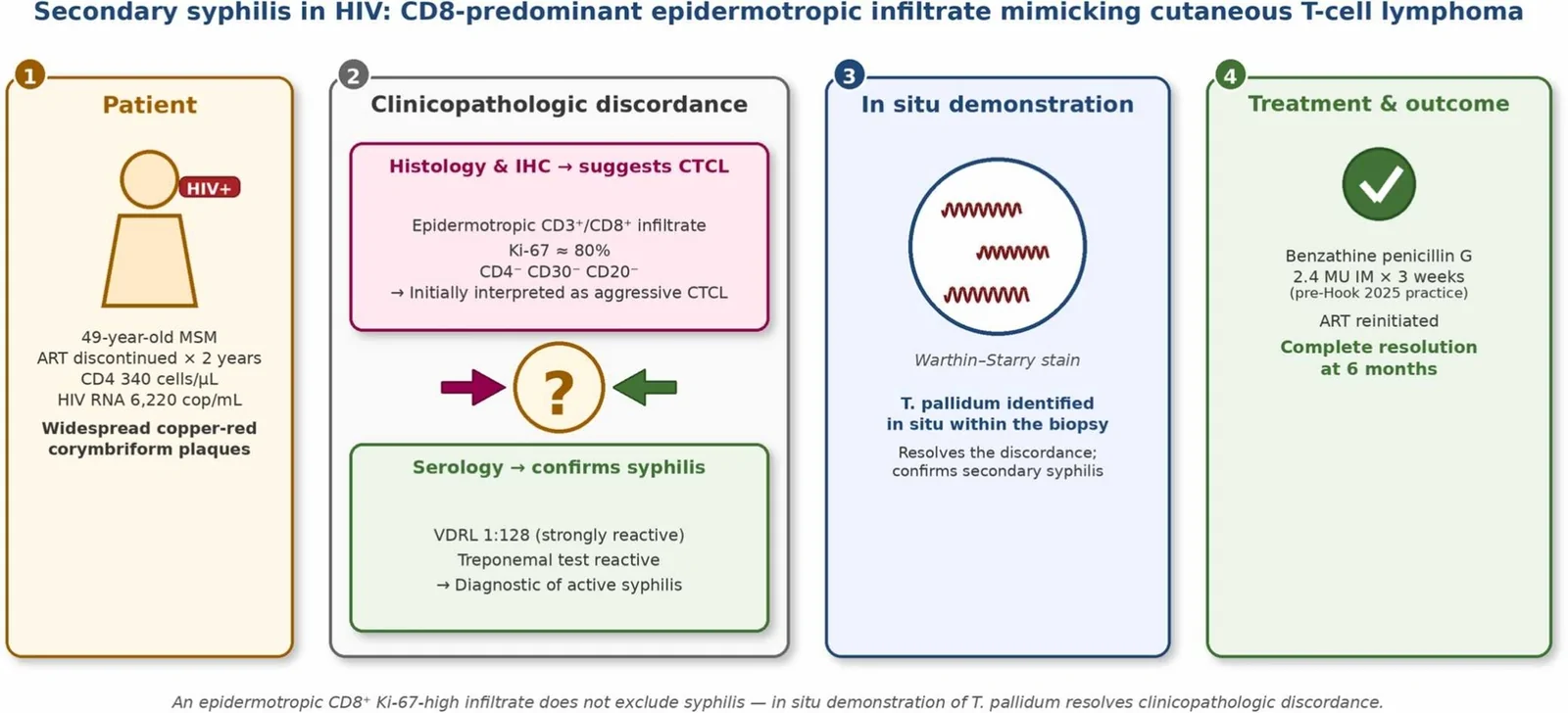

## Introduction

Syphilis, caused by *Treponema pallidum*, has resurged worldwide over the past decade. Although the highest incidence continues to be reported in men who have sex with men (MSM) and people living with HIV (PLWH), recent surveillance also documents a sustained rise in heterosexual adults and women of reproductive age, with a parallel increase in congenital syphilis [1,2]. Two entities that share the label “great imitator” of skin disease — secondary syphilis and mycosis fungoides (MF), the most common cutaneous T-cell lymphoma (CTCL) — converge in the differential diagnosis of atypical skin eruptions [[3], [4], [5]]; in PLWH the challenge is amplified because both reactive pseudolymphomatous responses and true cutaneous lymphomas are over-represented [[6], [7], [8], [9], [10]]. We describe a case in which strongly reactive syphilis serology was initially dismissed as an incidental coinfection because the histopathologic and immunophenotypic findings — CD8-predominant epidermotropism with high Ki-67 — anchored the diagnostic reasoning toward an aggressive epidermotropic CD8⁺ CTCL. In situ demonstration of *T. pallidum* ultimately resolved this clinicopathologic discordance. We highlight the histologic and immunophenotypic features that argue for and against CTCL versus syphilis, the role and diagnostic accuracy of direct-detection methods (anti-treponemal IHC, Warthin–Starry stain, lesional PCR), and the current recommended penicillin regimen for early syphilis in PLWH in light of recently published high-level evidence.

## Case presentation

A 49-year-old MSM with a 13-year history of HIV infection presented with an 8-kg weight loss over one month and a diffuse cutaneous eruption. He had discontinued antiretroviral therapy (ART) two years earlier; laboratory evaluation showed an HIV-1 viral load of 6220 copies/mL and a CD4 count of 340 cells/µL. Physical examination revealed multiple coalescing erythematous–violaceous macules and papulonodular plaques with a characteristic copper-red hue; several lesions were thickened and infiltrated, and some displayed a corymbriform-like configuration with a central larger plaque surrounded by satellite papules. The eruption began on both upper extremities and the posterior trunk and spread over three months to the left buttock and lower extremities, sparing the anterior trunk, face, palms, soles, and mucous membranes (Fig. 1). Generalized lymphadenopathy was not detected; ophthalmologic examination ruled out ocular involvement. The patient denied neurologic symptoms; cerebrospinal fluid analysis was offered but not performed in the absence of clinical neurologic findings.

**Fig. 1:**
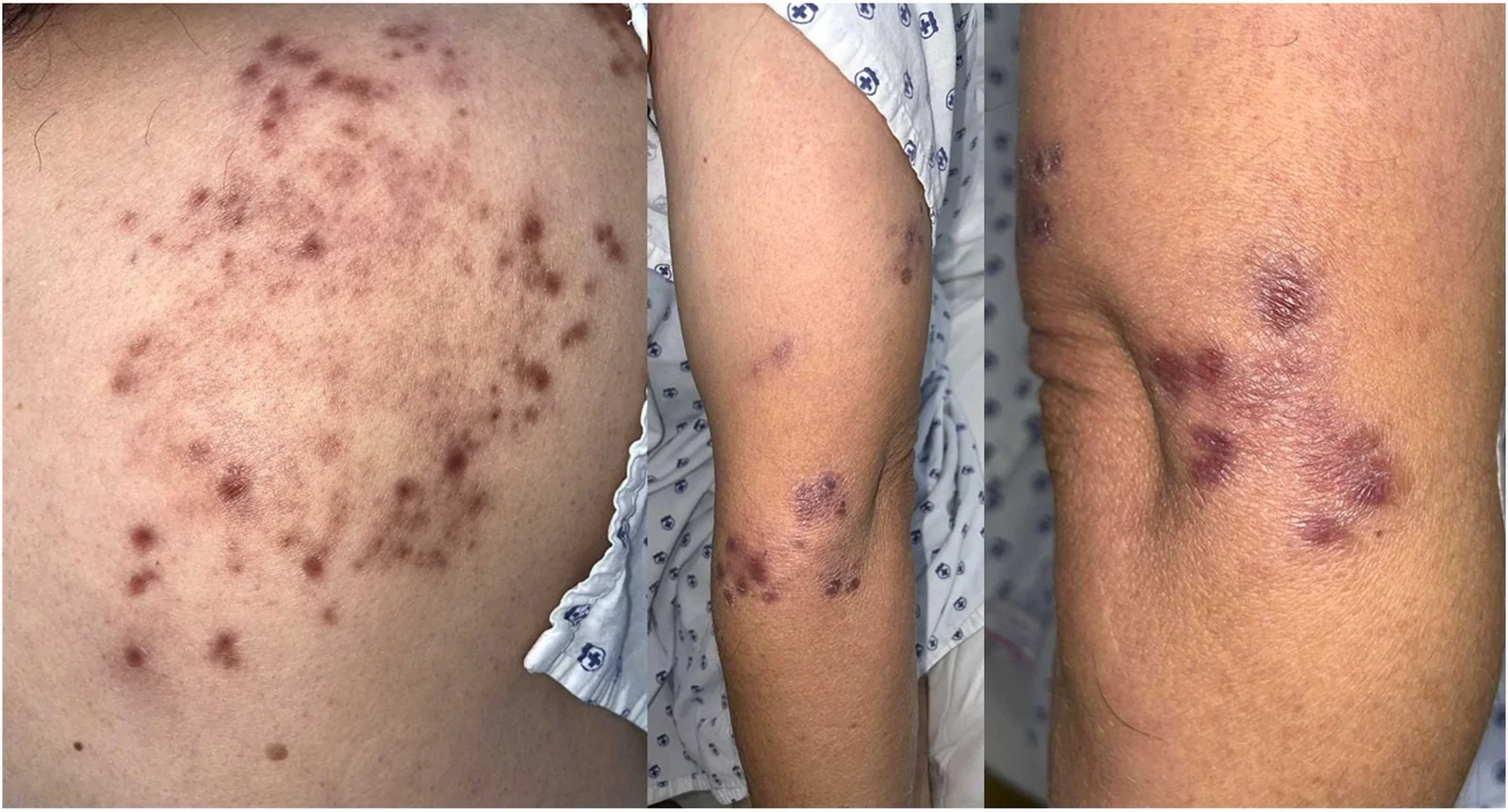
Coalescing erythematous–violaceous macules, papules, and infiltrated plaques with a characteristic copper-red hue and a corymbriform-like configuration on the posterior trunk and upper extremities.

Because widespread infiltrated eruptions in a host with poorly controlled HIV encompass a broad differential diagnosis — including cutaneous lymphomas (primary and secondary), Kaposi sarcoma, opportunistic infections (deep mycoses, mycobacterial disease, bartonellosis), lues maligna, and drug reactions, which cannot be resolved by serology alone — a targeted skin biopsy was ordered together with syphilis serology and HIV staging as part of the initial diagnostic work-up.

The nontreponemal test (VDRL) was strongly reactive at a titer of 1:128, and the treponemal test was reactive. Quantitative post-treatment titers were not retrievable from the laboratory record. Skin biopsy demonstrated acanthosis, superficial and deep lichenoid perivascular and perianexal lymphohistiocytic inflammation with admixed plasma cells, endothelial swelling and vascular proliferation, and isolated multinucleated giant cells. Immunohistochemistry showed CD3⁺ and CD8⁺ predominance with a Ki-67 proliferation index of approximately 80%, and was negative for CD20, CD4, CD30, ALK, and CD138, raising suspicion for an aggressive epidermotropic CD8⁺ CTCL.

Although the reactive VDRL of 1:128 was diagnostic of active syphilis, the histopathologic and immunohistochemical findings initially dominated the diagnostic reasoning, and the serologic result was interpreted as an incidental coexisting infection rather than as the cause of the eruption. Recognition of features atypical for aggressive CD8⁺ CTCL — the presence of plasma cells, endothelial swelling, absence of rapidly growing ulcerated necrotic nodules, and a sub-acute rather than fulminant course — prompted us to establish a definitive *in situ* etiologic link between the spirochete and the cutaneous infiltrate. Because polymerase chain reaction (PCR) for *T. pallidum* on lesional skin and anti-treponemal immunohistochemistry were not available at our institution, a Warthin–Starry silver stain was performed on the same biopsy, which identified scattered spirochetes morphologically consistent with *T. pallidum* (Fig. 2). The diagnosis of secondary syphilis with cutaneous involvement was confirmed.

**Fig. 2:**
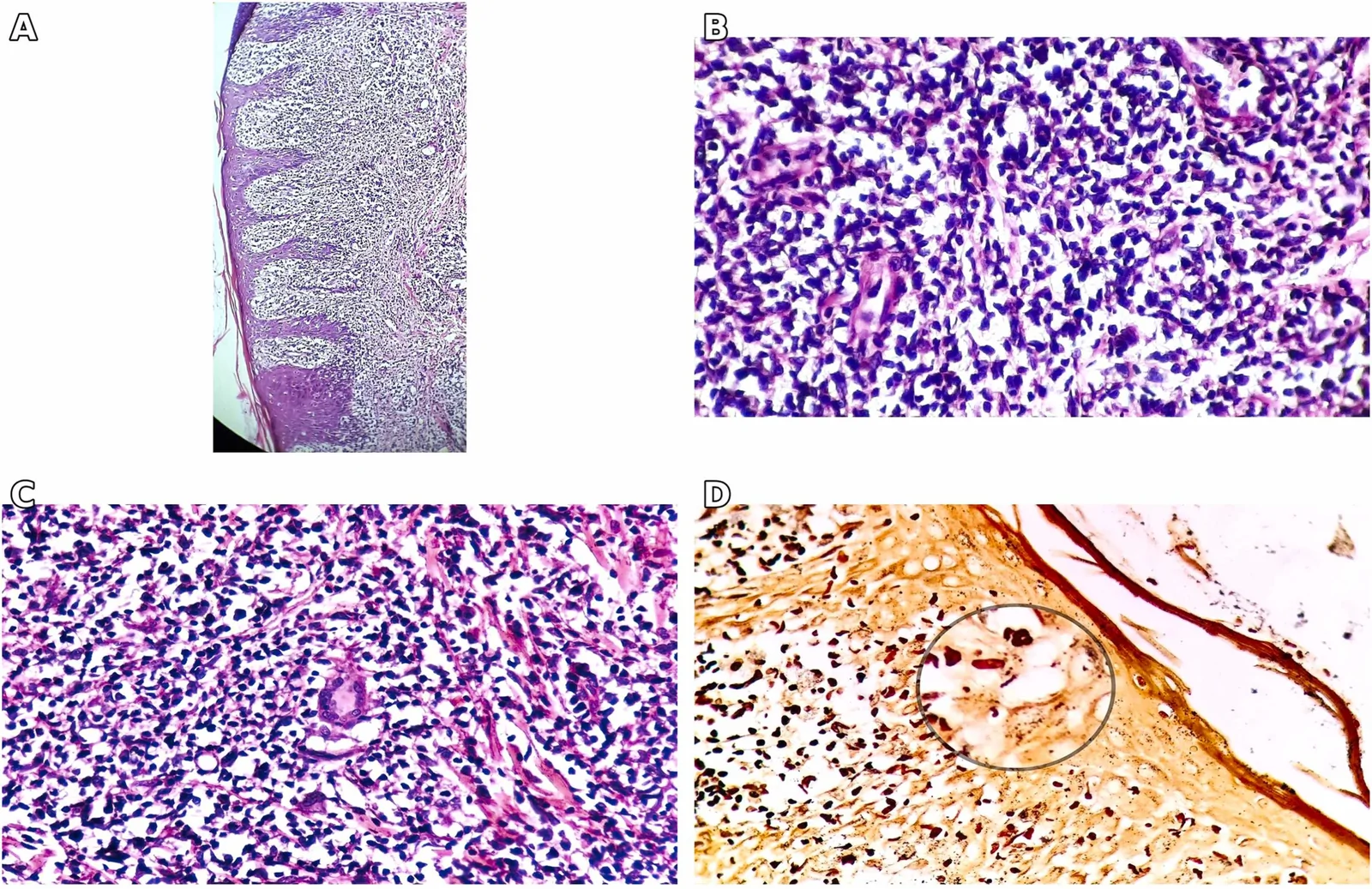
Skin biopsy. (A) Acanthosis with superficial and deep lichenoid perivascular and perianexal lymphohistiocytic inflammation, including admixed plasma cells (hematoxylin–eosin, original magnification ×5). (B) Endothelial swelling and vascular proliferation (hematoxylin–eosin, ×40). (C) Isolated multinucleated giant cells (×40). (D) Scattered spirochetes morphologically consistent with *Treponema pallidum* within the dermal infiltrate (Warthin–Starry stain, ×40).

The patient received intramuscular benzathine penicillin G 2.4 million IU weekly for three doses, in accordance with our institutional practice at that time for PLWH with extensive early syphilis (see Discussion), and antiretroviral therapy was reinitiated concurrently. Complete remission of the dermatosis was observed at six months after completion of treatment, leaving residual post-inflammatory hyperpigmentation (Fig. 3).

**Fig. 3:**
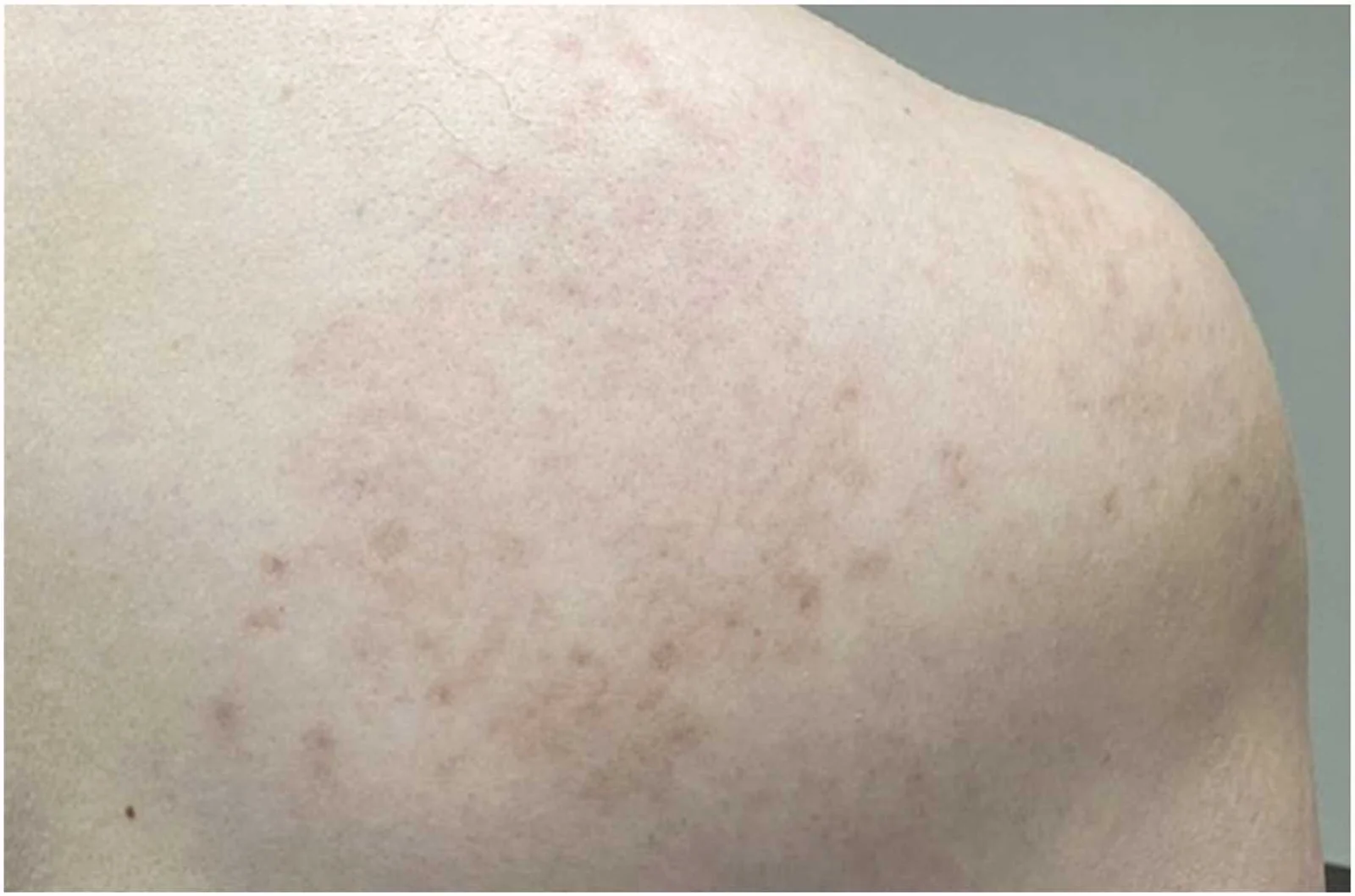
Posterior trunk six months after completion of intramuscular benzathine penicillin G, showing complete resolution of the active dermatosis with residual post-inflammatory hyperpigmentation.

## Discussion

### A clinicopathologic discordance, not an atypical syphilis

The educational value of this case is not that syphilis can mimic cutaneous lymphoma — that has been repeatedly documented [3,[6], [7], [8], [9], [10]]. The key lesson is that a set of striking histopathologic and immunophenotypic findings (CD8-predominant epidermotropism with a Ki-67 of ≈ 80%) can divert diagnostic reasoning away from a strongly supportive syphilis serology, and that this discordance can be resolved only by in situ demonstration of the spirochete. Our patient's presentation was in fact clinically and histopathologically characteristic of secondary syphilis — copper-red corymbriform papules with a plasma-cell-containing lichenoid infiltrate — and the diagnosis was unequivocally supported by a VDRL titer of 1:128 and a reactive treponemal test. The pitfall was cognitive anchoring on the immunophenotype, compounded by the high prevalence of asymptomatic syphilis seropositivity in MSM and PLWH, which occasionally leads clinicians to interpret reactive serology as coincidental rather than as the unifying diagnosis.

### Histopathologic and immunophenotypic features arguing for and against CTCL versus syphilis

Several features of our biopsy supported an infectious rather than neoplastic etiology. Plasma cells in a superficial and deep lichenoid infiltrate are considered a hallmark of syphilitic skin lesions and are typically absent in MF and in aggressive epidermotropic CD8⁺ CTCL (AECTCL) [6,9,11]. Endothelial swelling and vascular proliferation reflect the vasculotropism of *T. pallidum* and are not features of CTCL. The presence of isolated multinucleated giant cells further supports a reactive granulomatous response typical of syphilis. Clinically, our patient lacked the rapidly growing ulcerated tumors with central necrosis, aggressive systemic course, and multi-organ dissemination that define AECTCL [5,11].

Nevertheless, several features initially favored CTCL. Epidermotropism, CD8 predominance, and a high Ki-67 index are all recognized in AECTCL. Importantly, however, all three can occur in syphilitic pseudolymphoma, particularly in PLWH: Yamashita et al. [9] described an HIV-positive patient with secondary syphilis whose biopsy showed a CD8-predominant epidermotropic infiltrate initially interpreted as primary cutaneous CD8⁺ T-cell lymphoma; Mitteldorf et al. [10] documented a CD8-positive pseudolymphoma in lues maligna and HIV in which monoclonal T-cell receptor-β rearrangement was demonstrable, underscoring that clonality studies alone cannot discriminate reactive from neoplastic infiltrates. Maci et al. [8] recently emphasized that the absence of plasma cells — together with rapid, ulcerated, necrotic lesions and constitutional deterioration — should prompt greater caution before an oncologic diagnosis. In our case the presence of plasma cells, endothelial swelling and giant cells, the sub-acute course, and the copper-red corymbriform morphology were the decisive clues that redirected the differential toward syphilis. Table 1 summarizes the clinical, histopathologic, immunophenotypic and serologic features that argue for or against each entity.

**Table 1:** Differential diagnostic framework: secondary syphilis vs. cutaneous T-cell lymphoma (MF and AECTCL), including histopathologic and immunophenotypic features that argue for or against each entity.

**Feature**	**Secondary syphilis**	**Mycosis fungoides (early CTCL)**	**Aggressive epidermotropic CD8⁺ CTCL (AECTCL)**
Typical clinical presentation	Generalized maculopapular eruption with copper-red hue; palmoplantar involvement; corymbriform/nodular variants in HIV (lues maligna)	Slowly progressive, scaly erythematous patches and plaques on sun-protected areas; stages over years	Rapidly growing ulcerated nodules and tumors with central necrosis; aggressive course; poor prognosis
Mucosal/systemic clues	Mucous patches, condylomata lata, lymphadenopathy, constitutional symptoms, hepatitis, ocular or neurologic involvement	Usually skin-limited at early stages; lymph node involvement only at advanced disease	Frequent visceral spread; central-nervous-system involvement reported
Histopathology	Lichenoid/perivascular and perianexal lymphohistiocytic infiltrate; plasma cells often present (may be sparse in HIV/lues maligna); endothelial swelling and vascular proliferation; multinucleated giant cells occasional	Epidermotropic CD3⁺/CD4⁺ T-cells, Pautrier microabscesses, atypical cerebriform lymphocytes; plasma cells characteristically absent	Marked epidermotropism by atypical CD3⁺/CD8⁺ lymphocytes with high Ki-67; necrosis common; plasma cells absent
Immunohistochemistry	Reactive T-cell infiltrate (may be CD8⁺ predominant in HIV); polyclonal in most cases; T. pallidum demonstrable by anti-treponemal IHC (highest sensitivity), Warthin–Starry stain, or lesional PCR	CD3⁺/CD4⁺ predominance; loss of CD7; clonal TCR rearrangement	CD3⁺/CD8⁺/CD7⁻; βF1⁺/TCRγδ⁻; Ki-67 very high; clonal TCR rearrangement
Serology / microbiology	Reactive nontreponemal test (RPR/VDRL) confirmed by treponemal test (FTA-ABS, TPHA, CMIA); titers typically high in secondary disease	Not applicable	Not applicable
Features favoring syphilis over CTCL	Presence of plasma cells; endothelial swelling; multinucleated giant cells; absence of necrosis; corymbriform/copper-red morphology; sub-acute course; reactive high-titer serology; response to penicillin	—	—
Features that may falsely favor CTCL	Marked epidermotropism; CD8⁺ predominance; high Ki-67; occasional monoclonal T-cell receptor rearrangement in syphilitic pseudolymphoma	Progressive plaques resembling other dermatoses	Rapidly ulcerated tumors mimicking infectious granulomas
Response to therapy	Rapid clinical resolution after benzathine penicillin; serologic decline by ≥ 4-fold at 6–12 months	Skin-directed therapies; partial response with topical steroids in early MF	Aggressive multi-agent chemotherapy; allogeneic SCT considered; poor response

MF, mycosis fungoides; AECTCL, aggressive epidermotropic CD8⁺ cytotoxic T-cell lymphoma; CTCL, cutaneous T-cell lymphoma; SCT, stem-cell transplantation; TCR, T-cell receptor.

### When and how to demonstrate T. pallidum in skin — accuracy of Warthin–Starry, anti-treponemal IHC and lesional PCR

In patients with reactive syphilis serology, a skin biopsy is not routinely required to confirm secondary syphilis when the clinical presentation is classic. Biopsy — with in situ detection of *T. pallidum* — should be reserved for cases with atypical morphology suggesting an alternative diagnosis, coexisting immunosuppression that broadens the differential, suspicion of lues maligna or malignancy, poor response to appropriate antibiotic therapy, or clinicopathologic discordance such as in our patient.

When in situ demonstration of the spirochete is indicated, the three available techniques have markedly different diagnostic performance. Warthin–Starry silver staining is inexpensive and widely available but has limited sensitivity (approximately 30–70%) and imperfect specificity, because argyrophilic material and other spirochetes can mimic *T. pallidum* [11,12]. Immunohistochemistry with polyclonal or monoclonal anti-*T. pallidum* antibodies is the most sensitive and specific histologic method (sensitivity 70–90%, specificity >95%) and should be considered the current histologic reference standard where available [[12], [13], [14]]. Polymerase chain reaction on lesional skin has a sensitivity (70–95%) and specificity around 95%, and is particularly useful when serology is discordant with the clinical picture or when direct staining is negative in a highly suspicious lesion [2,14]. In our institution only Warthin–Starry staining was available; had anti-treponemal IHC or lesional PCR been accessible, we would have preferred those techniques for greater diagnostic yield. A pragmatic approach is: use IHC (or PCR) as first-line direct-detection method whenever available, and reserve Warthin–Starry for settings in which those techniques are not accessible — always interpreting the stain in conjunction with clinical, histopathologic and serologic findings, since a negative Warthin–Starry does not rule out syphilis.

### Penicillin regimen — pre-2025 clinical equipoise and current evidence

Our patient received three weekly doses of benzathine penicillin G 2.4 MIU IM (total 7.2 MIU), which corresponds to the regimen recommended for late latent syphilis. At the time of treatment, however, our institutional practice for PLWH with extensive early syphilis was to administer a three-dose regimen, reflecting a decades-long clinical equipoise: Rolfs et al. [15] first documented lower serologic responses in HIV-coinfected patients treated with single-dose benzathine penicillin G, giving rise to the concept of “enhanced therapy”; observational cohorts (Ganesan et al. [16]) and the first randomized trial specifically in HIV-coinfected patients (Andrade et al. [17], n = 64) subsequently suggested non-inferiority of a single dose, but were limited by sample size or observational design. In line with this accumulating evidence, the CDC 2021 STI Treatment Guidelines [18] formally recommend a single dose of benzathine penicillin G 2.4 MIU IM for early syphilis, including in PLWH; nevertheless, many HIV-focused clinics continued to administer three-dose regimens to patients with uncontrolled HIV and extensive cutaneous disease. The recently published multicentre, randomized, controlled noninferiority trial by Hook et al. [19] (249 participants, 61% PLWH; N Engl J Med 2025) has now definitively demonstrated that a single 2.4-MIU dose is non-inferior to three weekly doses for serologic response at six months, both overall and in the HIV subgroup. Following publication of this trial our institutional practice has been updated, and a single 2.4-MIU dose of benzathine penicillin G is now the standard regimen we use for early syphilis in PLWH.

## Conclusion

Secondary syphilis may present in PLWH with an epidermotropic CD8-predominant infiltrate and a very high Ki-67 index that closely mimics aggressive cutaneous T-cell lymphoma. The presence of plasma cells, endothelial swelling and multinucleated giant cells, together with the absence of ulcerated necrotic tumors and a sub-acute course, should raise diagnostic suspicion for syphilis and prompt in situ demonstration of *T. pallidum* — preferably by anti-treponemal immunohistochemistry or lesional PCR, or by Warthin–Starry when those are unavailable. A strongly reactive nontreponemal test in a patient with widespread cutaneous disease should be treated as the unifying diagnosis until directly disproven. In light of recent noninferiority evidence, a single 2.4-MIU dose of benzathine penicillin G is now the recommended regimen for early syphilis in PLWH; reinitiation of antiretroviral therapy is an integral component of management and, in our patient, led to complete clinical resolution.

## CRediT authorship contribution statement

**Aída S. Ramírez-González:** Conceptualization. **Felipe J. Bustos-Rodríguez:** Resources, Methodology. **Luz A. González-Hernández:** Resources, Methodology, Investigation. **Jaime F. Andrade-Villanueva:** Writing – review & editing, Project administration. **Luis H. Sat-Dávila:** Validation, Conceptualization. **Pedro Martínez-Ayala:** Writing – original draft, Formal analysis.

## Ethics declaration

Written informed consent to take part in the study and to publish the article has been obtained from all participants or their legal representatives. The privacy rights of participants have been observed.

This study was performed in compliance with relevant laws, regulatory frameworks and guidelines where the research took place. This study was conducted in accordance with Declaration of Helsinki (2013 revision); CARE guidelines for case reports; Mexican General Health Law – Reglamento en Materia de Investigación para la Salud. This study was approved by the Comité de ética en Investigación del Antiguo Hospital Civil de Guadalajara Fray Antonio Alcalde. (Approval No. CEI 68/26)

This research follows the CARE guidelines and the CARE checklist.

## Ethics approval

The study was conducted in accordance with the Declaration of Helsinki and was approved by the Ethics Committee in Research of the Antiguo Hospital Civil de Guadalajara “Fray Antonio Alcalde” (Registro de Investigación CEI 68/26, approved on 25 February 2026; oficio HCG/FAA/CEI-375/25).

## Consent to participate and consent for publication

Written informed consent for participation in this case report and for publication of clinical photographs and case details was obtained from the patient. A copy of the signed consent form is on file with the corresponding author and available on request.

## Patient consent

Written informed consent for participation and for publication of clinical photographs was obtained from the patient; the signed consent form is on file and available on request.

## Declaration of Generative AI and AI-assisted technologies in the writing process

A generative AI assistant (Claude, Anthropic) was used solely for English-language grammar and style correction during manuscript preparation. All scientific content, case description, figure preparation, interpretation of findings, and conclusions originated from and were verified by the named authors, who take full responsibility for the accuracy and integrity of the manuscript.

## IRB approval

Antiguo Hospital Civil de Guadalajara “Fray Antonio Alcalde” (protocol code CEI 68/26), approved on 25 February 2026.

## Funding

This research received no external funding.

## Declaration of Competing Interest

The authors declare that they have no known competing financial interests or personal relationships that could have appeared to influence the work reported in this paper.

## Data Availability

All data supporting the findings of this case are contained within the article.
